# Supplementary material for: Prolyl tRNA Synthetase Is Required for Mammarenavirus Multiplication
Source: Viruses. 2026 Feb 4;18(2):202. doi: 10.3390/v18020202 (PMC12944994; doi:10.3390/v18020202)
Supplement: Supplementary file 1 [file viruses-18-00202-s001.zip › viruses-4081332-supplementary.pdf]

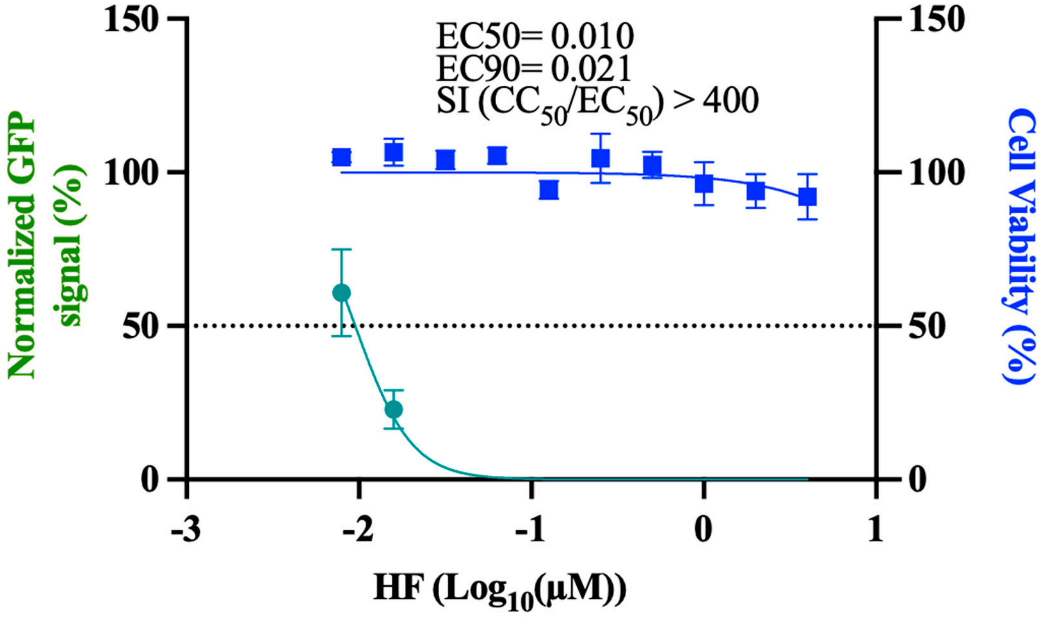

Figure S1. Dose-dependent effect of HF on LCMV multiplication in endothelial cells. Ea.hy926 cells were seeded at 4 × 10[4] cells/well into a 96-well plate, infected (MOI = 0.05) with rLCMV/WT-GFP-P2A-NP and treated with HF at the indicated concentrations. At 48 h pi, cells were fixed with 4% PFA, and numbers of infected cells determined by IF. Numbers of infected cells were normalized (%) to those of VC-treated samples cells and expressed as a % of infected cells (A). Cell viability was estimated based on DAPI staining signal quantified using the Cytation 5 reader. Results correspond to the average of four biological replicates. EC_{50} and CC_{50} values were calculated using a variable slope (based on four parameters) model and EC90 values were calculated using FindECanything model (logEC_{50}=logECF - (1/HillSlope)*log(F/(10-F)) with F parameter set to 10 (Prism10). Results show the mean and SD of four biological replicates.

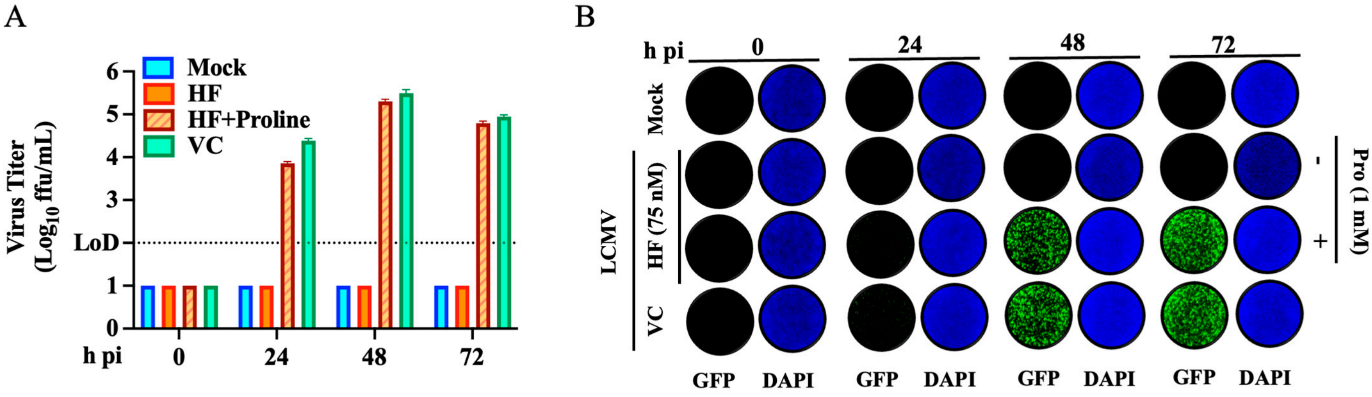

Figure S2. Biological replicate of HF (75 nM) on LCMV multi-step growth kinetics and peak titers in A549 cells. A. The counter effect of L-proline (Pro) on the production of infectious viral progeny in presence of HF. A549 cells were seeded at 5 × 10[5] cells/well in an M12-well plate, infected with rLCMV/GFP-P2A-NP (MOI 0.05), and treated with HF (75 nM), Pro (1 mM), the combination of HF and Pro, or with VC. At the indicated time points, cell culture supernatants were collected, and the titers of infectious virus were determined by the focus-forming assay (FFA) using Vero E6 cells. **B.** At the indicated h pi, samples from A were fixed with 4%PFA and washed with DPBS, sealed and stained with DAPI at the end of the experiment and imaged at 4x magnification using Keyence BZ-X710 series.

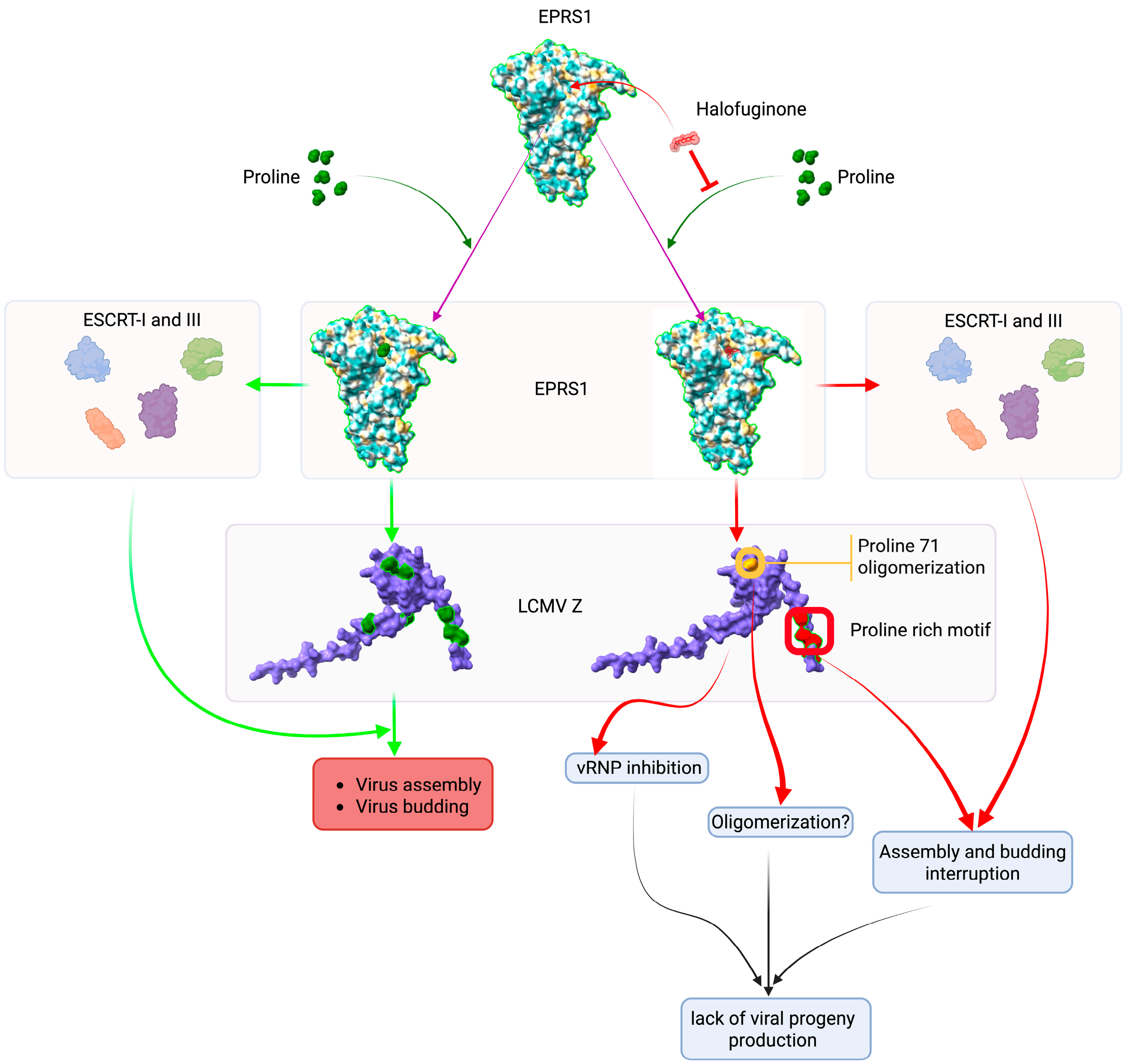

Figure S3. Proposed model of the effect of the prolyl tRNA synthetase 1 (PRS1) inhibitor, halofuginone (HF), on mammarenavirus assembly and budding. The PRS1 domain of the bifunctional enzyme glutamyl-prolyl-tRNA synthetase (EPRS1) catalyzes the incorporation of proline into both cellular and viral proteins. The mammarenavirus Z matrix protein orchestrates the viral assembly and budding processes. Proline-riche late domains critically contribute to Z budding activity via their interaction with components of cellular ESCRT machinery. Inhibition of PRS1 by HF disrupts Z-mediated viral assembly and budding processes, thereby blocking virus multiplication. HF-mediated inhibition of PRS1 inhibition also interferes with the activity of ESCRT-I and III components, which further contributes to disrupt Z-mediated budding. EPRS1 is depicted based on the PDB structure 4k88 [80]; LCMV Z matrix protein is presented as predicted using AlphaFold 3 server [81]. Red colored residues indicate the proline-rich motif late domains of Z matrix protein; orange residue corresponds to proline at position 71 implicated in Z oligomerization. The 3D lure was generated using ChimeraX v1.10.1. The protein sizes' are not for scale.
